# Influences of listeners' native and other dialects on cross-language vowel perception

**DOI:** 10.3389/fpsyg.2014.01065

**Published:** 2014-10-07

**Authors:** Daniel Williams, Paola Escudero

**Affiliations:** ^1^Area of Excellence – Cognitive Sciences, Linguistics Department, University of PotsdamPotsdam, Germany; ^2^The MARCS Institute, University of Western SydneySydney, NSW, Australia

**Keywords:** non-native speech perception, native dialects, non-native dialects, speech production, acoustic phonetics

## Abstract

This paper examines to what extent acoustic similarity between native and non-native vowels predicts non-native vowel perception and whether this process is influenced by listeners' native and other non-native dialects. Listeners with Northern and Southern British English dialects completed a perceptual assimilation task in which they categorized tokens of 15 Dutch vowels in terms of English vowel categories. While the cross-language acoustic similarity of Dutch vowels to English vowels largely predicted Southern listeners' perceptual assimilation patterns, this was not the case for Northern listeners, whose assimilation patterns resembled those of Southern listeners for all but three Dutch vowels. The cross-language acoustic similarity of Dutch vowels to Northern English vowels was re-examined by incorporating Southern English tokens, which resulted in considerable improvements in the predicting power of cross-language acoustic similarity. This suggests that Northern listeners' assimilation of Dutch vowels to English vowels was influenced by knowledge of both native Northern and non-native Southern English vowel categories. The implications of these findings for theories of non-native speech perception are discussed.

## Introduction

Two models on non-native speech perception, Best's (1995) Perceptual Assimilation Model (PAM) and Escudero's (2005, 2009) Second-Language Linguistic Perception Model (L2LP), posit that non-native sounds are perceived by functionally monolingual listeners in terms of native phonological categories. Both models propose that listeners perceptually assimilate or map non-native sounds to native phonological categories to varying degrees, depending on the perceived phonetic similarity to the native sound. Both models also posit that the resulting perceptual assimilation patterns are indicative of listeners' discrimination of the non-native speech sounds and subsequent second-language learning [see PAM's extension to second-language (L2) learning in Best and Tyler, 2007].

While perceptual assimilation patterns are based on native phonology in both models, only the L2LP explicitly claims that dialectal variation in the production of speech sounds (expressed in differences in acoustic values and weights for different acoustic cues) has a profound effect on speech perception. Specifically, the L2LP model posits that individuals learn to perceive sounds in a way that is optimal for their specific speech environment or dialect, as explained below, which means that individuals who are speakers of Dialect X will be able to perceive the sounds and contrasts in that dialect more accurately than those in Dialect Y. The purpose of the present study is to test the L2LP model's claims regarding dialectal variation in the context of non-native speech perception. To this end, the acoustic similarity and perceptual assimilation of Dutch vowels to those in two dialects of British English—a Northern and Southern variety—are compared.

Escudero's (2005) L2LP model is rooted in a more general theoretical account of first (L1) language learning. The model proposes that speech perception is underpinned by the mapping of auditory dimensions, such as vowel formants or voice onset time, onto a finite number of phonological categories; these mappings collectively form a *perception grammar*. Phonological categories emerge from the mapping of auditory dimensions according to how they are used and integrated in a listener's native language or language variety (Escudero, 2009). That is, listeners will prefer those auditory dimensions that reliably differentiate the sounds in their speech production. Listeners maximize the probabilities of understanding speech by making perceptual decisions based on the intended message, which in the L2LP model is referred to as the *optimal perception hypothesis*. Consequently, listeners with differential early experiences of language will differ in how they perceive the same auditory events; this not only applies to listeners with different language backgrounds, e.g., Dutch, German, or Spanish, but also to listeners who learned different dialects of the same language, e.g., Southern vs. Northern British English.

The L2LP model considers five components that are crucial to L2 acquisition, namely (1) the L1 and L2 languages and/or varieties involved, (2) the initial state of learning, (3) the learning task, (4) L2 development, and (5) the end state (Escudero, 2005, 2009). The first component assesses optimal perception of the L1, as described above. When faced with an unfamiliar non-native language, the model assumes that listeners will perceive it as they would perceive native speech due to their perception grammars being geared to the optimal perception of speech in their native speech environments, such as a particular dialect of a language. Thus, the second component of L2 learning assumes that listeners' optimal perception of the L1 is equal to the *initial state* of optimal L2 perception. Hence patterns of perceptual assimilation of non-native speech sounds to native categories are of theoretical interest because subsequent L2 perception and the learning tasks involved in order to develop optimal L2 perception are based on this initial state. For instance, two non-native sounds that are assimilated to the same native category will be difficult to discriminate and therefore learn. Importantly, perceptual assimilation patterns may not be the same for listeners who share the same broad language background but have different native dialects due to differing optimal perception for different native varieties.

In line with the L2LP model's predictions, several recent studies have shown that dialectal variation influences native and non-native speech perception. For instance, listeners with the same language background exhibit differential perception for vowels produced in different dialects of the same non-native language (e.g., Escudero, 2005; Escudero and Chládková, 2010). Furthermore, Chládková and Podlipský (2011) found that naïve Moravian Czech and Bohemian Czech listeners perceived the vowels in the Dutch contrasts /i-I/ differently in terms of Czech vowel categories due to differences in how their native Czech /iː-I/ contrast is produced. Such influences of particular native dialect have also been shown to pervade in L2 vowel perception. Escudero et al. (2012) found that listeners with either a North Holland Dutch or a Flemish Dutch dialect background categorized the English vowels /ε/ and /æ/ differently in terms of native Dutch vowels and this also correlated clearly with their L2 English vowel identification accuracy. Likewise, Escudero and Williams (2012) showed that Peruvian Spanish and Iberian Spanish L2 learners of Dutch identify some Dutch vowels differently, leading to subtle differences in their L2 vowel discrimination accuracy.

Dialectal variation is known to affect the perception of speech sounds in other dialects of the same language, especially if the sound belongs to a phonological category not present in listeners' native dialects (e.g., Dufour et al., 2007). Indeed, dialects of Northern British English generally lack the vowel /ᴧ/ as a separate phonological category and this has been shown to play a significant role in their speech perception of native and non-native dialects. Evans and Iverson (2004) found that Northern and Southern listeners who live in the South of England shift their exemplars on the F1 dimension for the vowels in the words “bud” and “cud,” both of which contain /ᴧ/ in Standard Southern British English (SSBE) but /ʊ/ in Sheffield English (SE), thereby mirroring the higher and lower F1 frequencies with which these vowels are produced in SSBE and SE, respectively. Despite the shift, the best exemplar locations of SSBE /ᴧ/ by Northern listeners in the South of England did not match how this vowel is produced by SSBE speakers, suggesting SSBE /ᴧ/ had not been stored accurately in long-term memory. Northern listeners who had lived in the North of England for all their lives, on the other hand, failed to display any such shift, suggesting that extensive exposure to SSBE gained by living in the South for several years was necessary to learn the new phonological category.

Conversely, dialectal variation may not have an apparent effect on speech perception. It has been shown that in difficult listening conditions, listeners—regardless of their native accent background—may show a bias toward standard dialects. For example, Clopper (2012) reports on a cross-dialect listening task in which listeners from three American English dialects were presented with sentences in noise said by talkers from four American English dialects and were asked to identify the final word. Listeners were most accurate at correctly identifying words in the General American dialect, a standard dialect, than any of the regional dialects, regardless of the listeners' own native dialects, suggesting a bias toward the standard in less favorable listening conditions. It was assumed that listeners' relative accuracy in General American was due to listeners' high familiarity with this dialect compared to regional dialects. It appears that, due to a high level of familiarity, listeners are able to show a degree of proficiency with a non-native dialect in their speech perception.

**Figure 1:**
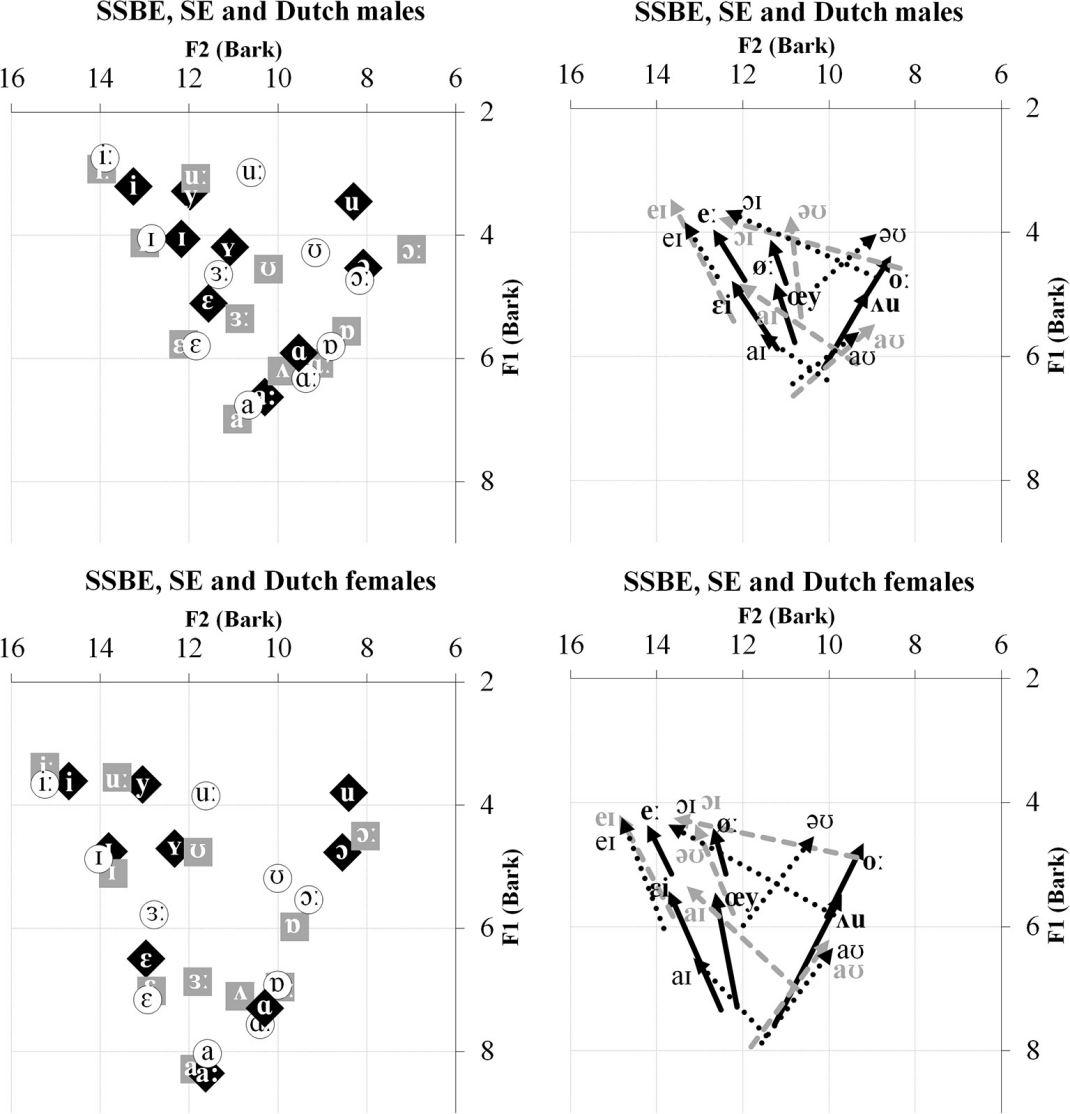
**Left:** Average F1 and F2 values for SSBE (gray squares), SE (unfilled circles), and Dutch (black diamonds) monophthongs produced by male and female speakers. **Right:** Average F1 and F2 trajectories of SSBE (gray dotted), SE (black dotted), and Dutch (thick solid black) diphthongs produced by male and female speakers.

The non-native language in the present study is Dutch; specifically, the standard variety of Dutch spoken in the Netherlands called Northern Standard Dutch. Excluding schwa, the Dutch vowel system has the nine monophthongs /i, y, I, Y, ε, aː, ɑ, ɔ, u/, and six diphthongs /eː, øː, oː, εi, œy, ᴧu/ (Collins and Mees, 2003)^1^. The two dialects of British English are SSBE and SE which are broadly representative of the major division of dialects in England into Northern and Southern English (e.g., as per Evans and Iverson, 2004). The vowel system of SSBE, excluding schwa, contains the 11 monophthongs /iː, I, ε, ɜː, a, ɑː, ᴧ, ɐ, ɔː, ʊ, uː/ and the five diphthongs /eI, əʊ, aʊ, aI, ɔI/ (McMahon, 2002), whereas SE's vowel system has the same five diphthongs but only 10 of the 11 monophthongs (Stoddart et al., 1999). Like many Northern English vowel systems, SE does not have a separate phonological vowel category for /ᴧ/, realizing vowels in English words that contain /ᴧ/ and /ʊ/ as [ʊ] (Wells, 1982; Williams and Escudero, 2014). Aside from this phonological difference, Williams and Escudero (2014) found a large number of acoustic differences between how the majority of SSBE and SE vowels are realized. For the monophthongs, SSBE and SE differ mainly in F1 and F2 values of /ɜː/ and all the back monophthongs /ɑː, ɐ, ɔː, ʊ, uː/. For the diphthongs /eI, əʊ, aI, ɔI/, SSBE generally exhibits greater amounts of formant movement than SE and, most strikingly, the directions of formant change of the diphthong /əʊ/ indicate it is phonetically forward closing in SSBE but backward closing in SE. Figure 1 displays average F1 and F2 values for the Dutch and two English dialects' monophthongs and diphthongs as produced by male and female speakers.

In order to predict listeners' perceptual assimilation patterns of non-native or second-language (L2) vowels to native categories, several recent studies have compared the phonetic similarity of vowels across languages by means of cross-language acoustic comparisons (for non-native, e.g., Nishi et al., 2008; Strange et al., 2009; Gilichinskaya and Strange, 2010; Escudero and Vasiliev, 2011; Escudero and Williams, 2011; Escudero et al., 2014; for L2, e.g., Escudero and Williams, 2012; Escudero et al., 2012). In many of these studies, cross-language acoustic similarity of vowels has been determined by means of discriminant analyses (DAs) which classify a set of non-native vowel tokens in terms of native vowel tokens (for a general overview, see Strange, 2007). The resulting classifications provide a quantitative way of determining the acoustically closest native vowel for each non-native vowel. Typically, DAs used to measure the cross-language acoustic similarity of vowels are performed on vowel token data comprising measurements along auditory dimensions relevant for perceiving vowels, such as duration and the frequencies of the first (F1), second (F2), and third (F3) formants as obtained at vowel midpoint to represent “vowel targets” of nominal monophthongs (e.g., Strange et al., 2007; Escudero et al., 2012). For instance, Gilichinskaya and Strange (2010) found that Russian listeners' perceptual assimilation patterns for seven out of eight American English monophthongs matched patterns of cross-language acoustic similarity obtained from DAs performed on midpoint F1, F2, and F3 values.

The success of DAs at predicting listeners' perceptual assimilation behavior depends on the input parameters and their relevance to speech perception. Escudero and Vasiliev (2011) suggest that employing formant frequencies at time points in addition to vowel midpoints enhances the consistency of classifications resulting from DAs and this is especially the case for the diphthongs or diphthongized monophthongs that are characteristic of several English dialects (Fox and Jacewicz, 2009). Moreover, dynamic spectral properties of vowels are relevant acoustic cues for the accurate identification of some monophthongs by native listeners of several English dialects (e.g., Nearey and Assman, 1986; Hillenbrand and Nearey, 1999; Iverson and Evans, 2007; Jacewicz and Fox, 2012).

The present study provides new evidence on the influence of native dialects on cross-language perception. According to the L2LP model, by comparing the acoustic properties of Dutch vowels with those of SSBE and SE vowels, it should be possible to make predictions about naïve Northern and Southern British English listeners' perceptual assimilation patterns of Dutch vowels to native vowels (Escudero, 2005, 2007, 2009). This study first addresses the general issue of whether cross-language acoustic similarity acts as a good predictor of listeners' perceptual assimilation patterns, as found in previous studies (e.g., Gilichinskaya and Strange, 2010; Escudero and Vasiliev, 2011; Escudero et al., 2012). Second, it investigates listeners whose dialects vowel systems differ phonologically and phonetically. While a previous study found different perceptual assimilation patterns by listeners from two Czech dialects for the non-native Dutch /i-I/ contrast (Chládková and Podlipský, 2011), the different phonological make-up of the SSBE and SE dialects' vowel inventories and the numerous acoustic differences between vowel categories are expected to generate several divergent patterns of cross-language acoustic similarity and perceptual assimilation. Finally, speakers of regional dialects in England, such as SE, will be highly familiar with the SSBE dialect, e.g., though the media (Stuart-Smith, 2007), while the opposite is less common. It is possible that SE listeners may have learned SSBE to some extent which may show in their speech perception (e.g., as per the American English listeners in Clopper, 2012). We thus also tested whether SE listeners' perceptual assimilation of Dutch vowels could have been influenced by this.

## Methodology

First, the cross-language acoustic similarity of Dutch vowels to those in SSBE and SE was gaged quantitatively by conducting analyses on corpora of Dutch and English vowel tokens. Second, SSBE and SE listeners participated in a perceptual assimilation task in which Dutch vowels were categorized in terms of native English vowel categories. If cross-language acoustic similarity is a good predictor of listeners' perceptual assimilation of non-native vowels, as the L2LP model proposes, then there should be a high correspondence between the two parts of the study.

### Cross-language acoustic similarity of vowels

#### Dutch and english corpora

The Dutch vowel tokens were produced by 22 Northern Standard Dutch speakers (11 female) reported by Van Leussen et al. (2011) and the SSBE and SE vowel tokens are those reported by Williams and Escudero (2014) produced by 17 SSBE speakers (10 female) and 19 SE speakers (10 female). The 58 speakers had a median age of 22 and an age range of 18–30. The 15 Dutch vowels /i, y, I, Y, ε, aː, ɑ, ɔ, u, e, ø, o, εi, œy, ᴧu/ came from monosyllables with the consonantal contexts /p_p/, /k_k/, /t_t/, /s_s/, and /f_f/ and the 16 English vowels /iː, I, ε, ɜː, a, ɑː, ᴧ, ɐ, ɔː, ʊ, uː, eI, əʊ, aʊ, aI, ɔI/ came from monosyllables with the consonantal contexts /b_p/, /g_k/, /d_t/, /s_s/, and /f_f/ ^2^. The corpora had been designed to provide acoustic information on canonical vowel production, i.e., “full” vowels in stressed syllables, in a range of stop and fricative environments. Phonetically voiced consonants were excluded to avoid possible vowel lengthening and to ensure vowel tokens could be reliably isolated in the digitized waveform (note that English initial /b, g, d/ tend to be produced without phonetic voicing in English). The monosyllables were produced in carrier sentences and two repetitions of each unique sentence were recorded.

For every vowel token, duration (ms) and formant (F1, F2, F3) frequencies (Bark) at three time points (25, 50, and 75% duration) were obtained following the procedure reported in Van Leussen et al. (2011). In order to determine the degree of formant movement, which is important for acoustically and perceptually classifying both English nominal monophthongs and diphthongs (e.g., Jacewicz and Fox, 2012), trajectory length (TL) was calculated for each vowel token as the Euclidean distance (Bark) in a F1/F2 space between F1 and F2 measurements at the 25 and 75% time points. As the direction of formant movement in the production of /əʊ/ differs markedly in SSBE and SE as described above, a measure of trajectory direction (TD) was calculated for each nominal diphthongs token as the angle (in degrees) of the diphthong trajectory in the F1/F2 space (as per Jin and Liu, 2013).

#### Discriminant analyses

In order to determine the acoustic similarity of Dutch vowels to those in SSBE and SE dialects, several DAs were run. For the nominal monophthongs (Dutch: /i, y, I, Y, ε, aː, ɑ, ɔ, u/ and English: /iː, I, ε, ɜː, a, ɑː, ᴧ, ɐ, ɔː, ʊ, uː/), acoustic data were included for duration, F1, F2 and F3 values (Bark) at 50% and TL (Bark). For the diphthongs (Dutch: /e, ø, o, εi, œy, ᴧu/ and English: /eI, əʊ, aʊ, aI, ɔI/), the measures of duration, TL and TD (degrees) were used^3^. Acoustic data from each speaker's two repetitions was averaged, yielding one vowel token per speaker in each of the five consonantal contexts. The training sets consisted of either SSBE or SE vowel tokens and the test sets always comprised the Dutch tokens. There were separate SSBE and SE training sets for monophthongs and diphthongs due to the different acoustic measures and these were further separated by speaker gender. The resulting parameter weightings and centers of gravity from these DAs were used to classify separately the male and female Dutch speakers' vowel tokens in terms of either SSBE or SE vowels.

### Cross-language perceptual similarity of vowels

#### Listeners

In total, 20 SSBE listeners (10 female) and 20 SE listeners (10 female) completed the perceptual assimilation task. Listeners had a median age of 23 and an age range of 18–30 and reported no or little proficiency in languages other than English. All SE listeners had grown up in the County of South Yorkshire in the North of England and had lived in the city of Sheffield for most of their lives, while all SSBE listeners had grown up in the Home Counties region in the South East of England and were living in London at the time of testing.

#### Stimuli and procedure

The auditory stimuli were 300 naturally produced Dutch vowel tokens, representing a subset of tokens in the Dutch corpus outlined above, and were taken from recordings of 20 (10 female) of the 22 Dutch speakers. Only vowel tokens produced in the /f_f/ consonantal context were used because in bilabial contexts Dutch vowels tend to be produced with the most “canonical” acoustic values and are not, for instance, subject to “fronting” which is common for Dutch back monophthongs in coronal consonantal contexts (Van Leussen et al., 2011). For a similar reason, the /f_f/ context has been used in other cross-language speech perception studies (e.g., Elvin et al., 2014). Additionally, including tokens from other four contexts would have increased the number of stimuli and consequently the length of the task.

Listeners were familiarized with 16 English orthographic response labels corresponding to the 16 British English vowels /iː, I, ε, ɜː, a, ɑː, ᴧ, ɐ, ɔː, ʊ, uː, eI, əʊ, aʊ, aI, ɔI/ and proceeded to the experiment once satisfied they were adequately familiar with them. Listeners then performed a multiple-alternative forced-choice identification in a sound-attenuated booth. For this, they were told they were going to hear speech sounds cut from running speech produced by several different speakers on each trial and to click on the label for the particular speech sound they identified. Before the experiment began, listeners completed 15 practice trials to confirm that they knew the vowel-response options and to familiarize themselves with the nature of the stimuli and task. The next trial began 1.0 s after the click of the response from the previous trial and the order of the stimuli was randomized by the presentation software. After every 30 trials, listeners could take short breaks and resume when ready. Including the familiarization of the orthographic response labels, the experiment lasted approximately 20–25 min.

## Results

**Figure 2:**
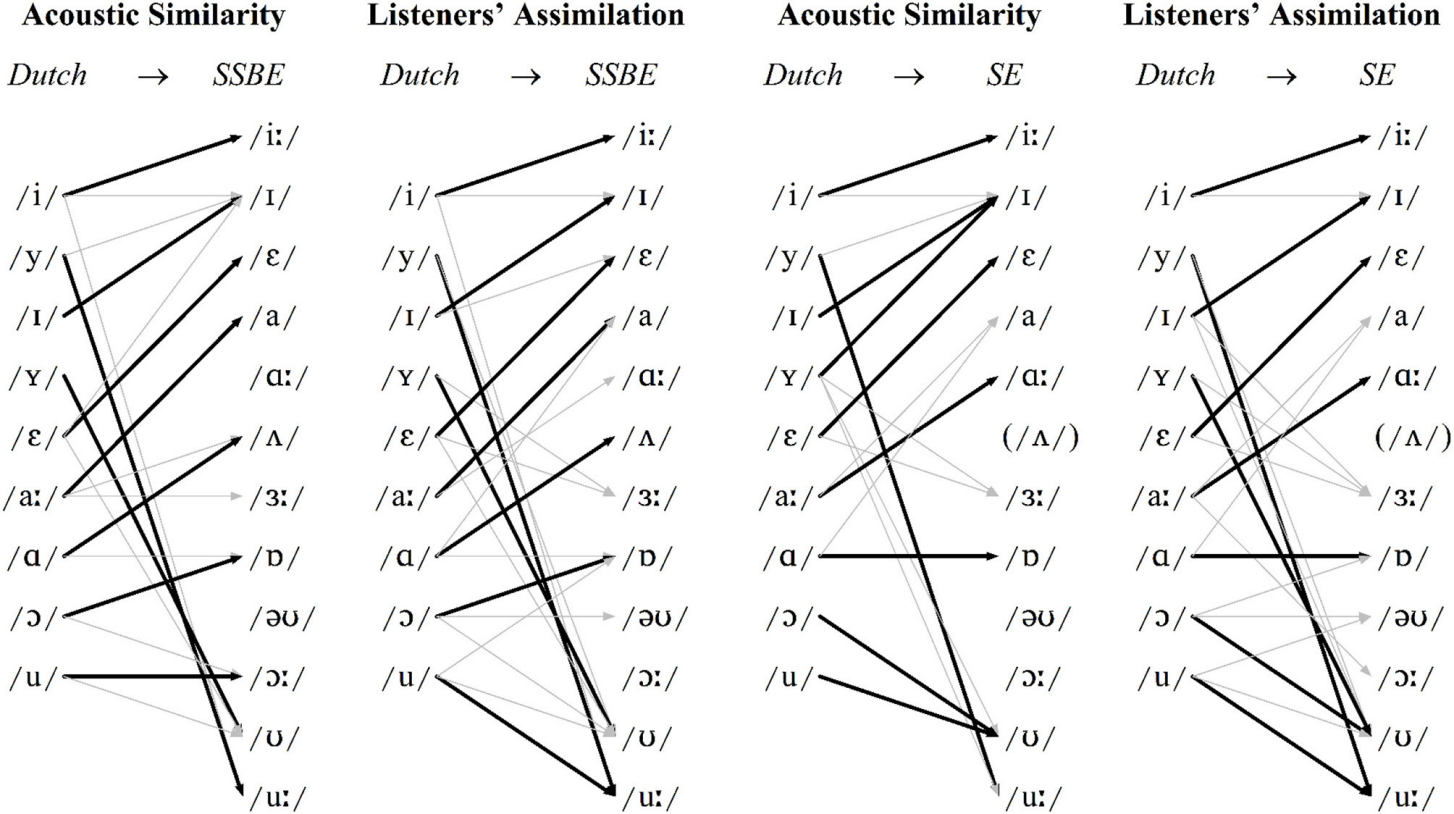
**Monophthongs: modal patterns of cross-language acoustic similarity and listeners' perceptual assimilation for SSBE (left) and SE (right)**. Modal classifications shown with black arrows and other classifications >10% shown with gray arrows.

**Figure 3:**
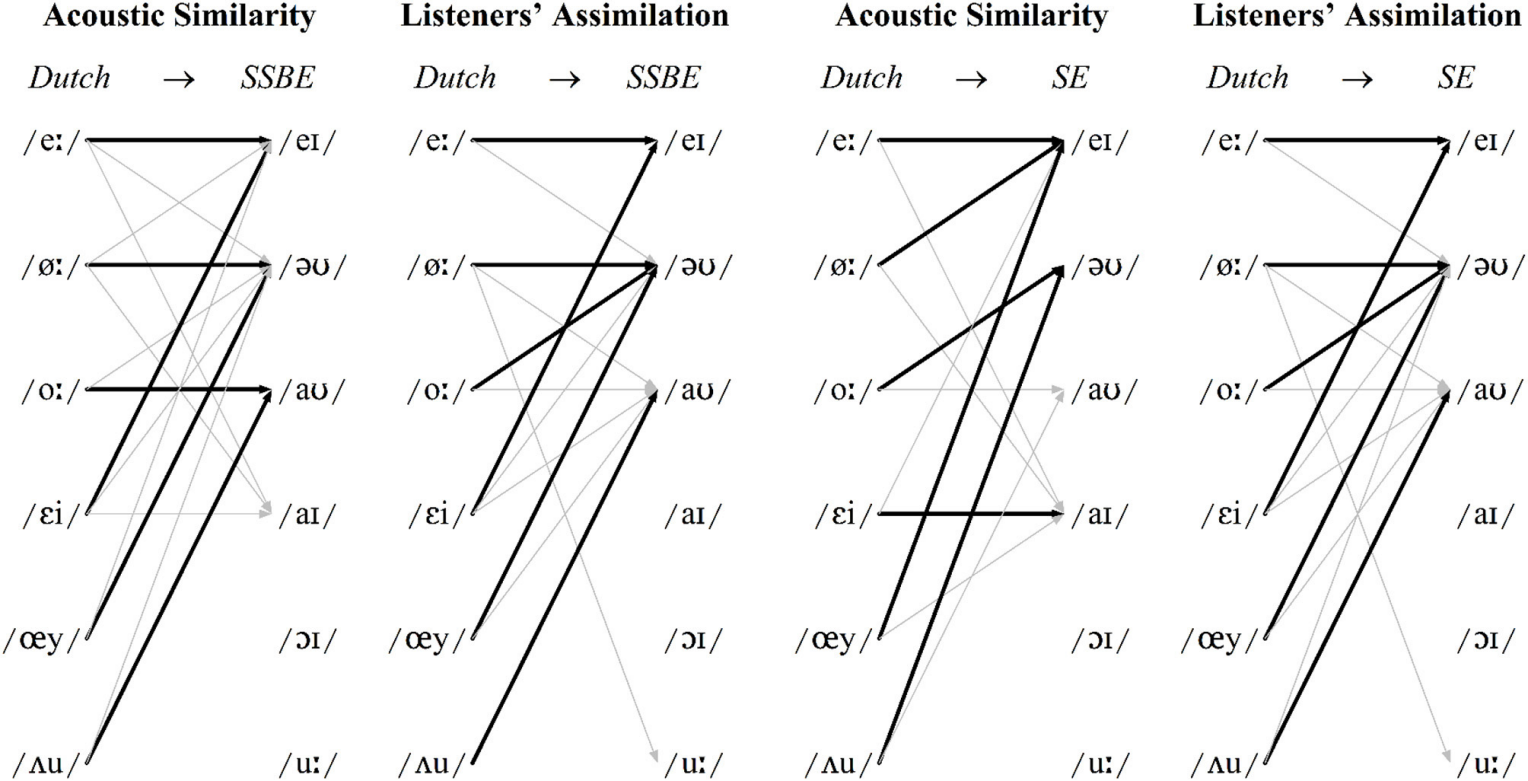
**Diphthongs: modal patterns of cross-language acoustic similarity and listeners' perceptual assimilation for SSBE (left) and SE (right)**. Modal classifications shown with black arrows and other classifications >10% shown with gray arrows.

In line with the results from Williams and Escudero (2014), as displayed in Figure 1, the greatest discrepancy between the two dialects' vowel systems is the presence or absence of the monophthong /ᴧ/. Also evident, the diphthong /əʊ/ moves in opposite directions in the F1/F2 space and there are several F1 and F2 differences between the non-back SE /ɐ, ɔː, ʊ, uː/ monophthongs and, to a lesser extent, the open vowels /a, ɑː/. Figures 2, 3 show the resulting classifications from the DAs, averaged across genders, which indicate cross-language acoustic similarity. Of particular interest are the modal classifications, i.e., the SSBE or SE vowel that a Dutch vowel was most often classified as, which are represented by solid black arrows, because these represent the acoustically closest vowel. More detailed results are provided in Table 2 in the Supplementary Material. Importantly, if listeners choose the same vowel category for the modal classification, then it can be said that acoustic similarity is a good predictor of perceptual assimilation patterns.

In the DAs, modal classifications made up 71 and 72% of SSBE and SE monophthong classifications, respectively. According to Figure 2, the four front three Dutch monophthongs /i, y, I, ε/ were classified most often in terms of the same English vowels, namely /iː, uː, I, ε/, respectively, and the remaining five Dutch monophthongs /Y, aː, ɑ, ɔ, u/ were classified most frequently in terms of different English vowels, namely SSBE /ʊ, a, ᴧ, ɐ, ɔː/, and SE /I, ɑː, ɐ, ʊ, ʊ/, respectively. The Dutch diphthongs shown in Figure 3 were less consistently classified, with modal classifications making up 66 and 65% of SSBE and SE diphthong tokens. While Dutch /eː/ is acoustically most similar to the same English vowel /eI/ in both SSBE and SE, the remaining five Dutch diphthongs were classified most frequently in terms of different English vowels.

It is unsurprising that the majority of Dutch vowels were classified as separate English vowel categories as both SSBE and SE exhibit large vowel systems like that of Dutch, meaning that multiple non-native vowels are less likely to be acoustically similar to a single English vowel. However, there are occasions, especially for diphthongs, when two Dutch vowels are acoustically most similar to a single English vowel, e.g., Dutch /eː-εi/ in SSBE, Dutch /øː-œy/ and Dutch /oː-ᴧu/ in both SSBE and SE.

Turning to the results of the perceptual assimilation task, Figures 2, 3 show that in addition to the modal response listeners chose one or two other responses to label most Dutch vowels. While this is not surprising as listeners had a choice of 16 response options, there is the question of whether the consistency of the two groups' categorization behavior is comparable. In order to address this, internal consistency scores were calculated for each listener, defined as the percentage of times their modal response was selected irrespective of the label (cf., Levy, 2009); averages are displayed in Table 1. Listeners' arcsine-transformed internal consistency scores were submitted to a repeated-measures analysis of variance (ANOVA) with Vowel as a within-subjects factor (15 levels for 15 Dutch vowels) and Dialect as between-subjects factor (two levels for two dialect groups).

**Table 1:** **SSBE and SE listeners' internal consistency scores (%) from the perceptual assimilation task**.

	**All Dutch vowels**	**Dutch monophthongs**	**Dutch diphthongs**
SSBE	62.0 (1.1)	57.7 (1.4)	68.5 (1.9)
SE	60.4 (1.2)	56.1 (1.4)	66.8 (2.0)
All listeners	61.2 (0.8)	56.9 (1.4)	67.7 (1.4)

*Standard errors shown in parentheses*.

The effect of Dialect did not reach significance and there was no significant Vowel ^*^ Dialect interaction (*p* > 0.05), confirming that SSBE and SE listeners selected their modal responses for each vowel with comparable frequencies. The analysis also yielded a main effect of Vowel [*F*_(14ε, 532ε, ε = 0.64)_ = 10.73, *p* < 0.0001], indicating that internal consistency scores differed per Dutch vowel. As Table 1 indicates, diphthongs were roughly 10% more consistently classified than monophthongs, which is unlike the cross-language acoustic similarity results in which diphthongs were less consistently classified. Listeners were clearly sensitive to diphthongization as the Dutch diphthongs were almost exclusively assimilated to native diphthongs. Lower internal consistency scores for monophthongs may thus be a result of the larger number of auditory stimuli and response options compared to diphthongs.

Importantly, Figures 2, 3 reveal only a partial correspondence between the classifications for cross-language acoustic similarity and listeners' perceptual assimilation patterns. The acoustic classifications indicate SSBE and SE listeners would perceive 10 of the 15 Dutch vowels in terms of different English vowel categories, namely the Dutch vowels /Y, aː, ɑ, ɔ, u, ø, o, εi, œy, ᴧu/. However, SSBE and SE listeners' modal responses in the perceptual assimilation task differed only for the Dutch monophthongs /aː, ɑ, ɔ/ and for none of the Dutch diphthongs. Apparently just three of the 10 predicted differences between SSBE and SE listeners' perceptual assimilation patterns were borne out.

As shown in Figure 2, SSBE modal classifications of eight of the nine Dutch monophthongs were the same in the DAs and perceptual assimilation task, while only the Dutch back rounded monophthong /u/ was assimilated to a different native SSBE vowel, namely /uː/ and not /ɔː/. As Figure 3 displays, of the six Dutch diphthongs, only Dutch /oː/ was perceived differently from the DA classification, namely as SSBE /əʊ/ instead of the acoustically closer SSBE /aʊ/. Overall, the SSBE acoustic predictions from the DAs were largely borne out in SSBE listeners' classifications.

According to Figure 2, the SE modal classifications were the same for seven of the nine Dutch monophthongs. This is because the front rounded Dutch monophthong /Y/, which is somewhat acoustically similar to /uː, ɜː, ʊ/, was assimilated by SE listeners most often to the SE back rounded monophthong /ʊ/, while the Dutch back rounded monophthong /u/, which is acoustically closest to the SE back rounded monophthong /ʊ/, was assimilated most often to SE /uː/. According to Figure 3, only two of the six Dutch diphthongs, namely /eː, oː/, were assimilated to the acoustically closest native vowel: while Dutch /øː, ᴧu/ are acoustically most similar to SE /eI, əʊ/, respectively, SE listeners assimilated these two Dutch diphthongs most often to SE /əʊ, aʊ/, respectively. Furthermore, although Dutch /εi, œy/ were found to be acoustically most like SE /aI/ and /eI/, respectively, listeners assimilated these Dutch diphthongs most often to SE /eI/ and SE /əʊ/, respectively. Thus, acoustic similarity predicted SE listeners' perceptual assimilation patterns for seven of the nine Dutch monophthongs and only two of the six Dutch diphthongs.

## Discussion

It appears that cross-language acoustic similarity is a much better predictor of SSBE listeners' perceptual assimilation patterns than those of SE listeners. This is because SE listeners' perceptual assimilation patterns often resembled those of SSBE listeners, which was not predicted by acoustic similarity as determined by the DAs.

It must be borne in mind that cross-language acoustic similarity was gaged in the DAs by keeping SSBE and SE tokens separate, thereby modeling SSBE and SE listeners' speech environments as being composed of vowel tokens produced exclusively by SSBE *or* SE speakers, respectively. Evidently, this did not provide an adequate baseline for predicting SE listeners' perceptual assimilation patterns for six of the 15 Dutch vowels (i.e., /Y, u, øː, εi, œy, ᴧu/). According to the L2LP model, knowledge of the phonetic properties of native phonological categories is determined by their distribution in listeners' speech environments. Hence the cross-language acoustic similarity of non-native to native speech sounds has been used to predict listeners' perceptual assimilation patterns because newly encountered and unfamiliar non-native speech sounds will be perceived in terms of those native categories found in listeners' speech environments (Escudero and Boersma, 2004; Escudero, 2005, 2006, 2009; Escudero et al., 2014).

Recent evidence demonstrates that knowledge of a non-native language (i.e., an L2) can be applied in perception of an L3 or other subsequently learned languages (L4, L5, LN). Escudero et al. (2013) found that Spanish learners of L3 Dutch with high proficiency in L2 English are much better able to learn new Dutch words differing in “difficult” vowel minimal pairs than Spanish learners with lower L2 English proficiency. The L2LP model accounts for this due to Spanish learners' proficiency in an L2 with a much larger vowel inventory than their L1. In learning Dutch as an L3, Spanish learners can copy their multiple L2 English vowel categories as a basis for learning L3 Dutch vowels. This is relevant to the present case as it is likely that SE listeners are highly familiar with SSBE, e.g., through its ubiquity in the media and in education (Stuart-Smith, 2007), even if SE individuals themselves do not produce many English vowels like SSBE speakers do (Williams and Escudero, 2014). It is possible that SE listeners based at least some of their responses on familiar SSBE sounds if this was a closer match than an SE equivalent or if listeners expected to hear SSBE if the language variety of the speakers was unknown (Clopper and Bradlow, 2008; Clopper, 2012).

**Figure 4:**
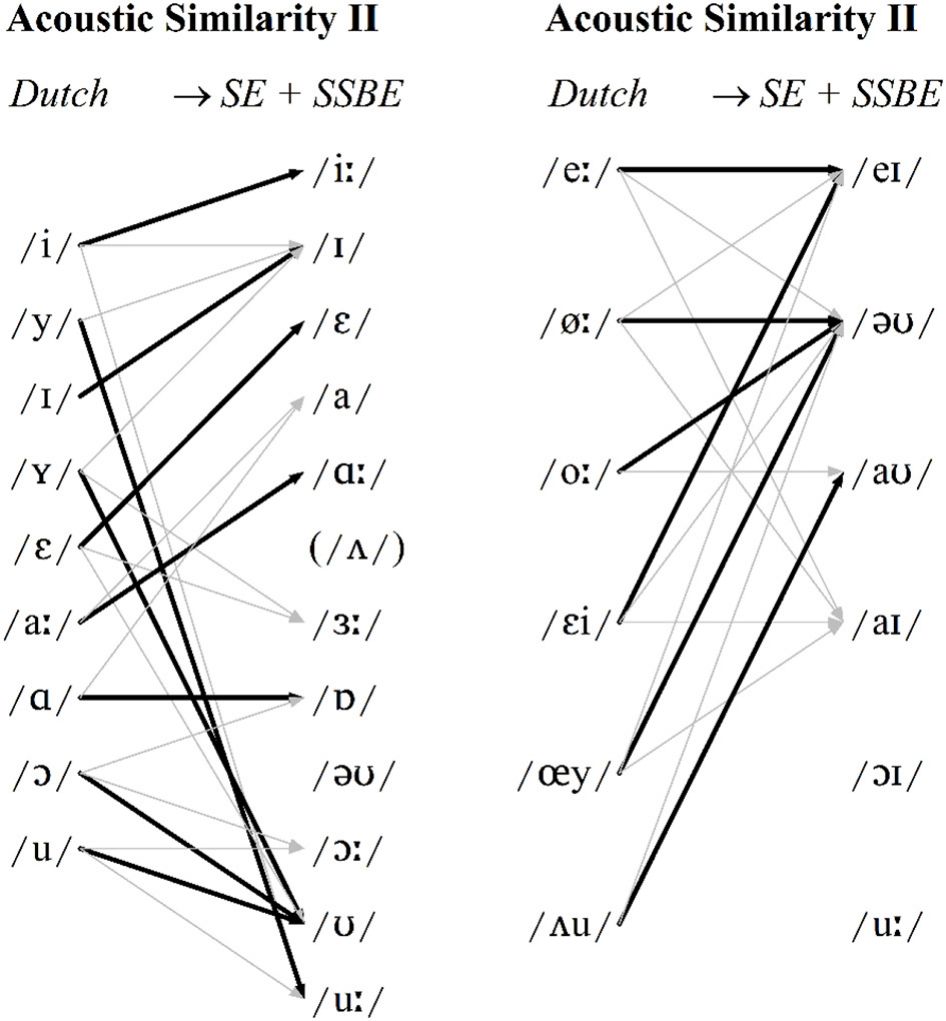
**Modal patterns of cross-language acoustic similarity when SSBE and SE tokens are combined for monophthongs (left) and diphthongs (right)**. Modal classifications shown with black arrows and other classifications >10% shown with gray arrows.

In light of Clopper (2012) and Clopper and Bradlow's (2008) studies, the proposal that SE listeners' perceptual assimilation patterns were influenced by the availability of a non-native was investigated. In order to do so, the cross-language DAs were re-run so that every Dutch vowel token was classified as *either* an SSBE *or* an SE vowel category—whichever was acoustically the closest. As in the previous analyses, vowel type (monophthong or diphthong) and speaker gender were kept separate but, unlike in the previous analyses, SSBE and SE tokens were coded as one of 30 vowel categories: two categories for each of the 15 vowels /iː, I, ε, ɜː, a, ɑː, ɐ, ɔː, ʊ, uː, eI, əʊ, aʊ, aI, ɔI/ (2 dialects × 15 vowels = 30 categories)^4^. The current DA was trained on all SSBE and SE tokens and tested with the same Dutch vowel tokens as before. The new classifications are displayed in Figure 4 with classifications collapsed across genders and the two English dialects for the 15 English vowels ^5^.

The new acoustic classifications in Figure 4 reveal a greater level of agreement with SE listeners' perceptual assimilation patterns displayed in Figures 2, 3. More detailed results are provided in Table 3 in the Supplementary Material. For instance, the Dutch monophthong /Y/ is now acoustically most similar to /ʊ/ and the acoustic similarity of the four Dutch diphthongs /øː, εi, œy, ᴧu/ to English vowels now match SE listeners' perceptual assimilation patterns of these to /əʊ, eI, əʊ, aʊ/, respectively. Importantly, the earlier differences found between SSBE and SE listeners' perceptual assimilation patterns of Dutch /aː, ɑ, ɔ/ are preserved in this second set of SE acoustic predictions. This is compatible with the L2LP model in which listeners may switch between perception grammars (the mappings of auditory dimensions onto speech sound categories) for different languages or language varieties depending on the listening context (Escudero, 2005, 2009). Specifically, the DAs containing both SSBE and SE vowel categories (Figure 4) effectively model the fact SE listeners may categorize a Dutch vowel token, e.g., /Y/, sometimes in terms of SSBE /ʊ/ but also sometimes in terms of an SE vowel such as /ɜː/.

The DAs in the present study failed to predict all SSBE and SE listeners' perceptual assimilation. This was the case for Dutch /u/, which as shown in Figure 1, has a strikingly lower F2 than either SSBE or SE /uː/—the modal classification for both listener groups. Interestingly, English /uː/ is undergoing or has recently undergone change in many dialects of British English, referred to as /uː/-fronting, in which /uː/ is produced with a relatively high F2 (Harrington et al., 2008). It thus seems likely that the young adult SSBE and SE listeners assimilated Dutch /u/ to English /uː/ due to their familiarity with older speakers' variants with a lower F2. Additionally, acoustic similarity did not predict SSBE listeners' assimilation of Dutch /oː/. As Figure 1 shows, SSBE /əʊ/ has a rising F2 contour which is also due to a recent sound change in the South of England which is replacing the older falling F2 (Kerswill and Williams, 2005) as displayed by Dutch /oː/. Cross-language acoustic similarity should thus include a range of commonly occurring variants in listeners' native speech environments.

The present study has found that the acoustic similarity of vowel productions can predict the perceptual assimilation of non-native vowels. Importantly, it has been shown that a non-native dialect can influence the perceptual assimilation of vowels in a non-native language. According to the L2LP model, this knowledge of vowels in native and non-native dialects reflects listeners' speech environments which may be wider than one particular dialect. This is especially the case for speakers of regional dialects who may be very familiar with the standard accent through regular exposure and therefore acquire substantial linguistic proficiency of this dialect. The L2LP model is compatible with this interpretation as it posits optimal perception of the speech signal based on the distributional properties of tokens in listeners' native speech environments, which is supported by findings from the influence of L2 in L3 learning (Escudero et al., 2013). Thus, when listening to speech sounds in an unfamiliar non-native language, listeners may switch between perception grammars—the mappings of auditory dimensions onto categories in native and non-native dialects or languages. In order to learn vowel inventories of other dialects, such as SSBE, or languages, the L2LP model claims that SE listeners will initially *copy* (i.e., reuse) their existing native vowel categories from SE into a new perception grammar. That is, SE listeners will need to adjust how auditory dimensions of speech sounds are mapped onto phonological categories in order to cope with physically different SSBE vowel tokens that reflect the intended linguistic message of the speaker (Escudero, 2005, 2009).

Other models on native and non-native speech perception do not overtly account for the influence of other dialects. While Best's (1995) PAM does not make explicit claims in this regard, Best and Tyler (2007) do not rule out that contact with dialects can lead to perceptual changes. For listeners who are proficient in more than one language or variety, the L2LP model claims that a particular perception grammar is activated according to the “language mode” which is determined by the listening task faced by a listener at a particular point in time (Escudero, 2005, 2009). In order to identify non-native vowels, listeners may resort to their knowledge of *either* their native dialect *or* a non-native dialect or language which are stored in separate perception grammars. In the perceptual assimilation experiment in the present study, a particular language mode was not expressly prompted by the task because listeners were presented with no clues about the language or dialect of the speech signal. As previously stated, SE listeners might have expected to hear SSBE in a university laboratory setting, thereby activating their non-native SSBE perception grammar some of the time.

Further research is of course required to test native and non-native proficiency in particular dialects and, in turn, its effects in non-native speech perception. As in the present study, Clopper (2012) indirectly assessed dialect familiarity by controlling for listeners' residency before the age of 18. However, other factors could have contributed to the bias toward General American in that study and some reasons provided by Clopper (2012, p. 13) include age and amount of exposure, travel experience and experience though media. Testing familiarity or proficiency is more straightforward in cases of L2 and L3 learning, as in Escudero et al. (2013), as phonological categories in the different languages have different communicative purposes. In the case of dialects, there is a vast amount of overlap or equivalence in the communicative functions of phonological categories even if their physical realizations evidently differ. Neurophysiological methods may provide fruitful for this purpose (e.g., Conrey et al., 2005; Brunellière et al., 2009, 2011) and to further test the L2LP model's claims.

Both PAM and the L2LP model posit that listeners' perceptual assimilation patterns represent perceived phonetic (dis)similarity between native and non-native sounds, but only the latter model is explicit with regard to predicting assimilation patterns. The L2LP model examines phonetic similarity in terms of relationships between the acoustic or auditory properties relevant in the perception of speech sounds, as in the present study. In this way, the L2LP model can make predictions of listeners' perceptual assimilation patterns. Best's (1995) PAM, on the other hand, describes phonetic similarity in terms of relationships between “gestural constellations” of speech sounds, which encompass, e.g., various articulators and gestures of the larynx. As pointed out by Strange (2007, p. 38), gestural similarity is only described abstractly in PAM and has not yet been supported by measurements of actual articulation. Furthermore, gestural constellations of vowels sounds are yet to be provided in work on PAM. This ultimately makes it problematic to determine what articulatory parameters are important for describing the similarity of vowel sounds and, consequently, how perceptual assimilation patterns could be predicted within its framework. Nevertheless, PAM (and PAM-L2) and the L2LP model do have in common that perceptual assimilation patterns predict discrimination of non-native contrasts.

Finally, while the present study provides new evidence in supporting the L2LP model's claims, there are some limitations with respect to fully addressing them. Specifically, this study investigated listeners as groups rather than individuals, whereas the L2LP model's optimal perception hypothesis is centered on *individual* listeners and their specific speech environments and individual speech productions (Escudero, 2005, 2009; Mayr and Escudero, 2010). Naturally, within the groups of SSBE and SE individuals there is bound to be variation in speech environments, speech perception and speech production. In order to fully test the L2LP model's optimal perception hypothesis, individual listeners' speech environments (via their speech production) would have to be compared to their individual perceptual assimilation patterns. However, it was not possible to carry out such an analysis here because the speakers who provided the SSBE and SE vowel tokens for the DAs were not composed of all of the listeners in the perceptual assimilation task.

In sum, the present study has shown once more that acoustic similarity can largely predict perceptual assimilation patterns of vowels in cross-language speech perception. It has been demonstrated that perceptual assimilation patterns by listeners whose native dialect is a regional dialect may be influenced by their familiarity of more standard varieties. The L2LP model accounts for this proposal in terms of listeners being able to switch between perception grammars, i.e., how auditory dimensions are mapped onto speech sound categories which is largely determined by their native speech environments.

### Conflict of interest statement

The authors declare that the research was conducted in the absence of any commercial or financial relationships that could be construed as a potential conflict of interest.

## Footnotes

^1^Collins and Mees (2003) class the Dutch vowels /eː, øː, oː/ as “potential diphthongs.” Adank et al.'s (2004) acoustic description of Dutch vowels treats these three vowels as diphthongs and shows that they display significant formant change.

^2^Both SSBE and SE speakers were prompted to produce the nonsense words containing /ᴧ/ and /ʊ/ so that it could be determined whether speakers contrasted the two vowels in their speech.

^3^Mid-point formant measurements were not included in the DAs involving diphthong tokens as this measure represents “vowel targets” of the spectrally steady-state portions of monophthongs which diphthongs are not expected to exhibit.

^4^SSBE /ᴧ/ tokens were excluded a priori because SE listeners scarcely made use of this response option in the perceptual assimilation task (Figure 2) and also because SE listeners are unlikely to exhibit a distinct perceptual exemplars of this vowel unless they have lived in the South of England for several years as per the results of Evans and Iverson (2004).

^5^Results from each English vowel category are collapsed across SSBE and SE as they are functionally equivalent.
