# Supplementary material for: Influences of listeners' native and other dialects on cross-language vowel perception
Source: Front Psychol. 2014 Oct 7;5:1065. doi: 10.3389/fpsyg.2014.01065 (PMC4188024; doi:10.3389/fpsyg.2014.01065)
Supplement: Supplementary file 1 [file DataSheet1.DOCX]

**Supplementary material**

**Table 2. Classification of Dutch vowels in terms of SSBE (right) and SE (left) vowels by acoustic similarity measures and listeners’ perceptual assimilation patterns.** Percentage of time a particular vowel was chosen is given in parentheses. Results show the most frequently chosen category (“Modal”) and others chosen > 10% of the time (“Other >10%”).

| Dutch  Vowel | SSBE | | | | SE | | | |
| --- | --- | --- | --- | --- | --- | --- | --- | --- |
|  | Acoustic similarity | | Listeners’ assimilations | | Acoustic similarity | | Listeners’ assimilations | |
|  | Modal | Other >10% | Modal | Other >10% | Modal | Other >10% | Modal | Other >10% |
| i | iː (59) | ɪ (26), uː (15) | iː (58) | ɪ (21), uː (11) | iː (71) | ɪ (29) | iː (48) | ɪ (22) |
| y | uː (74) | ɪ (16) | uː (75) | ʊ (15) | uː (55) | ɪ (39) | uː (52) | ʊ (22) |
| ɪ | ɪ (94) | - | ɪ (50) | ɛ (13) | ɪ (97) | - | ɪ (42) | ɜː (13), ʊ (13) |
| ʏ | ʊ (92) | - | ʊ (53) | ɜː (20) | ɪ (32) | uː (26), ʊ (21), ɜː (21) | ʊ (44) | ɜː (23) |
| ɛ | ɛ (65) | ʊ (20), ɪ (12) | ɛ (56) | ɜː (12), ʊ (11) | ɛ (75) | ɜː (13) | ɛ (54) | ɜː (14) |
| aː | a (54) | ɜː (20), ʌ (20) | a (58) | ɑː (21) | ɑː (59) | a (41) | ɑː (34) | a (29), ɔː (13) |
| ɑ | ʌ (81) | ɒ (14) | ʌ (42) | ɒ (22), a (15) | ɒ (71) | a (17) | ɒ (37) | a (24) |
| ɔ | ɒ (59) | ɔː (35) | ɒ (50) | əʊ (15), ʊ (12) | ʊ (95) | - | ʊ (40) | əʊ (23), ɒ (22) |
| u | ɔː (64) | ʊ (26) | uː (52) | ʊ (19), ɒ (12) | ʊ (89) | - | uː (43) | ʊ (33), əʊ (12) |
| eː | eɪ (41) | əʊ (37), aɪ (22) | eɪ (52) | əʊ (18) | eɪ (55) | aɪ (45) | eɪ (52) | əʊ (16) |
| øː | əʊ (64) | eɪ (15), aɪ (12) | əʊ (58) | uː (17), aʊ (15) | eɪ (70) | aɪ (16) | əʊ (43) | aʊ (17), uː (14) |
| oː | aʊ (88) | əʊ (12) | əʊ (67) | aʊ (19) | əʊ (76) | aʊ (21) | əʊ (66) | aʊ (13) |
| ɛi | eɪ (51) | əʊ (30), aɪ (19) | eɪ (50) | əʊ (24), aʊ (17) | aɪ (52) | eɪ (45) | eɪ (47) | əʊ (22), aʊ (12) |
| œy | əʊ (69) | eɪ (19) | əʊ (63) | aʊ (28) | eɪ (75) | aɪ (22) | əʊ (43) | aʊ (42) |
| ʌu | aʊ (82) | əʊ (18) | aʊ (81) | - | əʊ (63) | aʊ (32) | aʊ (56) | əʊ (21) |

**Table 3. Classification of Dutch vowels in terms of English vowels by acoustic similarity measures.** Percentages shown are averages across common SSBE and SE vowel categories.

| Dutch  Vowel | Modal | Other >10% |
| --- | --- | --- |
| i | iː (47) | uː (33), ɪ (20) |
| y | uː (62) | ɪ (28) |
| ɪ | ɪ (84) | - |
| ʏ | ʊ (59) | ɪ (18), ɜː (14) |
| ɛ | ɛ (54) | ʊ (27), ɜː (11) |
| aː | ɑː (50) | a (36) |
| ɑ | ɒ (65) | a (21) |
| ɔ | ʊ (73) | ɒ (15), ɔː (12 |
| u | ʊ (71) | ɔː (12), uː (11) |
| eː | eɪ (68) | aɪ (20), əʊ (12) |
| øː | əʊ (48) | eɪ (33), aɪ (15) |
| oː | əʊ (72) | aʊ (27) |
| ɛi | eɪ (56) | aɪ (25), əʊ (18) |
| œy | əʊ (58) | eɪ (26), aɪ (14) |
| ʌu | aʊ (53) | əʊ (41) |
